# Activation of α2A-adrenergic signal transduction in chondrocytes promotes degenerative remodelling of temporomandibular joint

**DOI:** 10.1038/srep30085

**Published:** 2016-07-25

**Authors:** Kai Jiao, Guang Zeng, Li-Na Niu, Hong-xu Yang, Gao-tong Ren, Xin-yue Xu, Fei-fei Li, Franklin R. Tay, Mei-qing Wang

**Affiliations:** 1 State Key Laboratory of Military Stomatology, Department of Oral Anatomy and Physiology and TMD, School of Stomatology, Fourth Military Medical University, 145 Changle Western Road, Xi’an, 710032, China; 2 Department of Dentistry, Tangdu Hospital, Forth Military Medical University, Shannxi, Xi’an, 710038, China; 3 State Key Laboratory of Military Stomatology, Department of Prosthodontics, School of Stomatology, Fourth Military Medical University, Changle Western Road No.145, Xi’an, 710032, China; 4 Undergraduate Department of Oral Science, Fourth Military Medical University, Changle Western Road No.145, Xi’an, 710032, China; 5 State Key Laboratory of Military Stomatology, Department of Orthodontics, School of Stomatology, Fourth Military Medical University, 145 Changle Western Road, Xi’an, 710032, China; 6 The Dental College of Georgia, Augusta University, Augusta, GA, USA

## Abstract

This study tested whether activation of adrenoreceptors in chondrocytes has roles in degenerative remodelling of temporomandibular joint (TMJ) and to determine associated mechanisms. Unilateral anterior crossbite (UAC) was established to induce TMJ degeneration in rats. Saline vehicle, α2- and β-adrenoreceptor antagonists or agonists were injected locally into the TMJ area of UAC rats. Cartilage degeneration, subchondral bone microarchitecture and the expression of adrenoreceptors, aggrecans, matrix metalloproteinases (MMPs) and RANKL by chondrocytes were evaluated. Chondrocytes were stimulated by norepinephrine to investigate signal transduction of adrenoreceptors. Increased α2A-adrenoreceptor expression was observed in condylar cartilage of UAC rats, together with cartilage degeneration and subchondral bone loss. Norepinephrine depresses aggrecans expression but stimulates MMP-3, MMP-13 and RANKL production by chondrocytes through ERK1/2 and PKA pathway; these effects were abolished by an α2A-adrenoreceptor antagonist. Furthermore, inhibition of α2A-adrenoreceptor attenuated degenerative remodelling in the condylar cartilage and subchondral bone, as revealed by increased cartilage thickness, proteoglycans and aggrecan expression, and decreased MMP-3, MMP-13 and RANKL expressions in cartilage, increased BMD, BV/TV, and decreased Tb.Sp in subchondral bone. Conversely, activation of α2A-adrenoreceptor intensified aforementioned degenerative changes in UAC rats. It is concluded that activation of α2A-adrenergic signal in chondrocytes promotes TMJ degenerative remodelling by chondrocyte-mediated pro-catabolic activities.

Osteoarthritis is a major cause of chronic disability and affects nearly 27 million people in the USA alone^1^. Current therapies are not adept at impeding or reversing the cartilage degeneration and subchondral bone change associated with osteoarthritis progression^2^. The sympathetic nervous system plays crucial roles in bone development, metabolism and remodelling^3^. Although articular cartilage is avascular and devoid of nerve innervation, recent studies showed that high levels of norepinephrine, the major sympathetic neurotransmitter, were detected in the synovial fluid of patients with joint trauma^4^; those patients have increased incidence to develop post-traumatic osteoarthritis^5^. Sprouting of sympathetic nerve fibres was identified in subchondral bone during the early stage of osteoarthritis, extending into the overlying cartilage via vascular channels^6^,^7^. Despite these observations, the role of the sympathetic nervous system in the initiation and progression of osteoarthritis remains obscure.

The paracrine effects of norepinephrine are mediated via the adrenoreceptor family comprising α1, α2 and β subtypes; each subtype is further classified into three isoforms^3^. Although adrenoreceptors are constitutively expressed in chondrocytes from different origins, the effect of receptor activation on chondrocyte metabolism is controversial^8^. Analyses of growth plate chondrocytes indicate that β-adrenergic signals suppress differentiation of chondrocytes by decreasing type II collagen and Indian hedgehog expression^8^,^9^,^10^,^11^, and inhibit their hypertrophic differentiation by decreasing type X collagen and matrix metalloproteinase-13 (MMP-13) expression and chondrocyte apoptosis^8^,^9^,^12^. Jenei-Lanzl *et al*. reported that β-adrenergic signal transduction in cartilage progenitor cells of knee osteoarthritis patients accelerated their hypertrophic differentiation by accelerating type X collagen and MMP-13 expression, and inhibiting type II collagen and glycosaminoglycans production^4^. Conversely, Lorenz *et al*. reported that similar transduction of β-adrenergic signals reversed interleukin (IL)-1β-induced reduction in type II collagen and glycosaminoglycans expression and augmentation in IL-8 and MMP-13 expression^13^. Whereas β-adrenergic signals suppressed the cell cycle and proliferation of osteoarthritic chondrocytes, α-adrenergic signals increased proliferation and induced apoptosis of those cells^13^. A dual function of norepinephrine in osteoarthritic chondrocytes was thus proposed: β-adrenergic signalling plays an anti-inflammatory role in osteoarthritis pathogenesis by promoting a stable articular chondrocyte phenotype to counteract cartilage degradation, whereas α-adrenergic signalling promotes cartilage degradation through induction of chondrocyte apoptosis^13^. However, the longitudinal expression profile of adrenoreceptors by articular chondrocytes during osteoarthritis development, and the reversal effects of adrenoreceptor antagonists or agonists on osteoarthritis progression are unknown.

Disrupted balance of the cartilage extracellular matrix (ECM) occurs in osteoarthritis due to decreased synthesis of the ECM components by chondrocytes, and excess production of matrix-degrading enzymes^14^. MMP-3 degrades most of the cartilage ECM components and activates MMP-9^15^. MMP-13 is the most potent degrading enzyme of type II collagen; its over-expression in chondrocytes causes cartilage degradation in osteoarthritis^16^. MMP-13 also degrades type I collagen, the primary organic component of bone ECM, and stimulates osteoclast differentiation^17^. Increased RANKL/OPG ratio in chondrocytes derived from the knee joint cartilage of osteoarthritis patients^18^ or experimental animals^19^, resulted in increased osteoclast activity and subchondral bone loss. This RANKL-induced osteoclastogenesis was further enhanced by MMP-13^17^. To date, it is unknown whether expressions of cartilage ECM components and degrading factors of the osteochondral complex by chondrocytes would be affected by the sympathetic tone during osteoarthritis progression.

The temporomandibular joint (TMJ) is one of the most common osteoarthritis sites^20^. Because abnormal mechanical loading is an important pathogenic factor in osteoarthritis development and the biomechanical conditions of TMJ are closely related to dental occlusion^2^, the authors have developed rodent models with TMJ degenerative remodelling through abnormal dental occlusion, named unilateral anterior crossbite (UAC)^19^,^21^,^22^,^23^,^24^,^25^. Degenerative remodelling in the rodent TMJs were characterised by progressive cartilage degradation, increased subchondral bone remodelling and osteochondral angiogenesis. Furthermore, sprouting of sympathetic nerve fibres and up-regulated norepinephrine levels were identified from the degenerative condylar subchondral bone of UAC rats^22^; these phenomena were probably responsible for cartilage degenerative remodelling via osteochondral interaction. In the present study, this UAC rat model was used to determine the longitudinal expression profile of chondrocytic adrenoreceptors, and the effects and underlying mechanism of norepinephrine on the anabolic and catabolic activities of chondrocytes were also examined. Furthermore, whether injection of adrenoreceptor antagonists or agonists into the local TMJ region could reverse/aggravate TMJ degenerative remodelling were also investigated.

## Materials and Methods

### Animal model

Six-week old female Sprague-Dawley rats (180–190 g) were obtained from the Animal Centre of the Fourth Military Medical University. All animal procedures were performed according to the guidelines of the Animal Care Committee of the Fourth Military Medical University, Xi’an, China, and all experimental protocols were approved by Fourth Military Medical University. In the experimental groups, a unilateral anterior crossbite prosthesis (UAC) was bonded to the lower incisors of each rat to induce abnormal mechanical loading on its TMJs^22^,^25^. Rats in the control groups underwent a mock operation procedure without permanent bonding of the unilateral anterior crossbite prosthesis. To pharmacologically manipulate adrenoreceptor signalling, the physiological saline (Veh), the selective α2-adrenoreceptor antagonist yohimbine (Yoh, 100 μg) or agonist clonidine (Cld, 10 μg), the selective β-adrenoreceptor antagonist propranolol (Pro, 10 μg) or agonist isoprenaline (Iso, 10 μg) were injected into the local areas of the bilateral TMJs of the experimental rats, respectively. The dosage employed for the aforementioned drugs (Sigma, USA) was based on previous reports^26^,^27^,^28^, as well as the authors’ pilot study. All rats received the same standardised diet and no rat showed any signs of disability during the experimental period.

### Temporomandibular joint local area injection

Fifty microlitre each of the aforementioned drugs diluted by physiological saline or the same volume of physiological saline (vehicle) was injected into the local TMJs regions of the experimental rat once every week, from the first day when the anterior crossbite prosthesis was inserted. The technique of the injection followed what was reported in our previous work^24^,^25^. Briefly, the needle of a specially made Hamilton-type syringe was inserted just below the zygomatic arch between the eye and ear until the outer surface of the mandibular ramus was reached. The orientation of the needle tip was adjusted to enable it to go along the bony wall to reach the TMJ region.

### Group designation and sampling

Rats from the control and experimental groups were sacrificed at the end of 2, 4 or 8 weeks of the experimental period. The drug-treated experimental rats were all sacrificed at the end of the 4-week experimental period. Because no differences in degrading changes were identified between the left side and the right side of the TMJs in experimental rats in the authors’ previous reports^22^,^25^,^29^, the left condylar tissue blocks were harvested, fixed, decalcified and embedded in paraffin for preparation of 5 μm-thick sagittal sections. The sections were used for histochemical and immunohistochemical stainings (N = 6). The right condyle were used for micro-computed tomography (GE eXplore Locus SP, London, United Kingdom) (N = 6). In addition, the left condylar cartilage from the other 15 rats were dissected and used for real-time polymerase chain reaction (RT-PCR) analysis, while the right condylar cartilage of those rats were used for western blot analysis. In each subgroup, every 3 out of 15 condylar cartilage were pooled together to create a single sample for RT-PCR and western blot analyses (N = 5).

### Histochemical staining and histomorphometry

Haematoxylin and eosin staining was used to evaluate histochemical changes within the condyles. Safranin O-fast green staining and toluidine blue staining were performed to determine changes in proteoglycans distribution within the condyles. Condyle cartilage thickness, the percentage area of safranin O and toluidine blue staining were measured as reported previously^25^. Briefly, the condylar cartilage was evenly divided into anterior, central and posterior third and the cartilage thickness in the central or posterior third was measured and averaged. The percentage area of proteoglycans distribution was calculated using the values of the safranin O or toluidine blue staining areas in the central and posterior third, divided by the value of the total area of the central and posterior third of the condylar cartilage, respectively.

### Micro-computed tomography

Trabecular microstructure and bone mineral density (BMD) of the condylar subchondral bone were analysed by micro-computed tomography as previously described^19^,^22^. Briefly, two cubes (0.5 × 0.5 × 0.5 mm each) were selected from the middle of the central and posterior third of the condylar subchondral bone. Within the selected regions, BMD, bone volume fraction (BV/TV), bone surface-to-volumeratio (BS/BV), trabecular thickness (Tb.Th), trabecular number (Tb.N) and trabecular separation (Tb.Sp) were determined using the MicroView Advanced Bone Analysis 2.1.2 software (GE Healthcare, Pittsburgh, PA, USA).

### Immunohistochemical staining

Tissue processing, section staining and counting of immune-positive cells were performed as reported previously^21^,^22^,^23^,^24^,^25^. The primary antibodies were goat polyclonal α2A-adrenoreceptor (1:75; sc1478, Santa Cruz Biotechnology, Inc., USA), rabbit polyclonal β2-adrenoreceptor (1:100, ab137494; Abcam, Cambridge, United Kingdom), rabbit polyclonal aggrecan (1:200, ab36861, Abcam), goat polyclonal MMP-3 (1:50, sc6839, Santa Cruz), MMP-13 (1:50, sc30073, Santa Cruz), RANKL (1:50, sc7628, Santa Cruz). Six squares were applied at the quartering points of the central (each 0.15 mm × 0.15 mm) and posterior (each 0.2 mm × 0.2 mm) third of the condylar cartilage. Within the selected frames, the number of immune-positive cells and the percentage area of aggrecan-positive staining were determined. In the isotype control slides, isotype antibodies were substituted for the primary antibodies.

### RT-PCR

Gene expression of adrenoreceptors and factors related to cartilage metabolism, such as aggrecan, type II collagen, type X collagen, MMP-3, MMP-9, MMP-13, RANKL and OPG were detected by RT-PCR as described previously^21^,^22^,^23^,^24^,^25^. Briefly, total RNA was extracted using Trizol (Thermo Fisher Scientific, Waltham, MA, USA). Primers for the target genes were listed in supplemental Table 1. Gene expression was analysed with the 7500 real-time PCR (Thermo Fisher Scientific), using glyceraldehyde 3-phosphate dehydrogenase (GAPDH) as the internal control. The amount of target cDNA relative to GAPDH was calculated using the 2^−ΔΔCT^ method. Results were calculated as the relative quantification compared to the control group, which was set at 1-fold. Data were collected from 3 independent pooled samples.

### Western blotting

Total protein from each group was fractionated by SDS-PAGE and transferred onto a nitrocellulose membrane. The membrane was blocked with 5% non-fat milk and incubated with primary antibodies against α2A-adrenoreceptor (1:200, sc1478, Santa Cruz), β2-adrenoreceptor (1:500, ab137494, Abcam), aggrecan (1:500, ab36861, Abcam), MMP-3 (1:200, sc6839, Santa Cruz), MMP-13 (1:300, sc30073, Santa Cruz), RANKL (1:300, sc7628, Santa Cruz), β-actin (1:1000, 3700, Cell Signalling Technology, USA), Phospho-ERK1/2 (Thr202/Tyr204) (1:800, 4370, Cell Signalling Technology), total-ERK1/2 (1:1000, 4695, Cell Signalling Technology), phospho-p38 (Thr180/Tyr182) (1:800, 4511, Cell Signalling Technology), total-p38 (1:1000, 9212, Cell Signalling Technology), phospho-JNK (1:800, 4688, Cell Signalling Technology), total-JNK (1:1000, 9252, Cell Signalling Technology), phospho-Akt (Thr308) (1:800, 4056, Cell Signalling Technology) and total-Akt (1:1000, 4691, Cell Signalling Technology). Signals were revealed by incubation with a horseradish peroxidase-conjugated secondary antibody (1:5000, Zhongshan Golden Bridge Biotechnology, China) and enhanced chemiluminescence detection^22^,^23^,^24^,^25^.

### Chondrocyte isolation from TMJ and norepinephrine stimulation

Chondrocytes were isolated from the TMJ condylar cartilage of six-week old female Sprague-Dawley rats by digestion with 0.25% trypsin (Sigma, USA) for 20 min, followed by digestion with 0.2% type II collagenase (Invitrogen, USA) for 2–3 h^22^. The isolated chondrocytes were re-suspended in DMEM medium (Gibco, USA) containing 10% fetal bovine serum (Hyclone, USA). Cells were then plated in 60-mm diameter plates at a density of 1.5 × 10^6^ cells/plate. After primary culture for 5 days, the chondrocytes were harvested. Secondary cultures were placed in 6-well plates at a density of 5 × 10^5^ cells per well for the following experiments, and two chondrocyte-specific markers, aggrecan and type II collagen, were detected by toluidine blue staining and immunofluorescence microscopy, respectively.

To observe the short-term effect of norepinephrine treatment, the chondrocytes were treated once with norepinephrine (N5785, Sigma) for 15 min, 30 min, 1 h, 4 h and 12 h at 10^−6^, 10^−7^ or 10^−8^ M concentration. To observed long-term effect of norepinephrine treatment, the chondrocytes were treated repeatedly with norepinephrine for 24 h, 48 h and 96 h at 10^−6^ or 10^−8^ M concentration. The incubation medium were changed every 4 h to ensure concentration consistency of norepinephrine. For short-term inhibition assay, 10^−7^ M norepinephrine were used to further confirm whether α- or β-adrenoreceptors is involved in the norepinephrine-induced pro-catabolic effects on chondrocytes, because 10^−7^ M norepinephrine has been reported to be capable of activating both α- or β-adrenoreceptors^13^. Chondrocytes were treated with 10^−7^ M norepinephrine for 1 h alone, or pre-treated for 1 h with doxazosin (D9815, Sigma, a selective α1-adrenoreceptor antagonist), yohimbine (Y3125, Sigma, a selective α2-adrenoreceptor antagonist), propranolol (P0884, Sigma, a non-selective β-adrenoreceptor antagonist), ICI 118,551 (0821, R&D Systems, USA; a selective β2-adrenoreceptor antagonist), U0126 (9903, a selective ERK1/2 inhibitor), SP600125 (8177, a selective JNK inhibitor), SB203580 (5633, a selective p38 inhibitor) or LY294002 (9901, a selective PI3K/Akt inhibitor), all at 10^−5^ M concentration. The inhibitor pre-treated chondrocytes were subsequently stimulated with 10^−7^ M norepinephrine for 1 h. All pathway inhibitors were obtained from Cell Signalling Technology, Inc. The inhibitor pre-treatment time and concentration were in accordance with the manufacturer’s instructions. For long-term inhibition, chondrocytes were treated 96 hours by physiological slaine vehicle, 10^−8^ NE, or 10^−8^ NE supplemented with different adrenoreceptor antagonists, and the incubation media were changed every 4 h.

### Statistical analyses

Cell counting measurements were performed in a blinded manner by two independent observers (RG, XX) using Photoshop CS7.0 software (Adobe Systems Corp., USA). Inter-observer reliability was analysed by calculating the Intraclass Correlation Coefficient (ICC) for the measurements^22^. There was a high level of agreement between the two observers (ICC = 0.914) and the average value of the two measurements from the same specimen was used for further statistical analysis. Data were expressed as means ± standard deviation for each group. Normality of data distribution was tested by Shapiro-Wilk test with 95% confidence and Levene’s test was used to assess homogeneity of variance. The assumptions for parametric tests were fulfilled and statistical significance among groups was evaluated by ANOVA. Post-hoc comparison between groups was performed using the Tukey test. P-values less than 0.05 were considered statistically significant for all tests.

## Results

### Body weight

All rats remained healthy during the course of the study and no significant differences in body weight were noticed between the experimental rats and their age-matched controls, and between the drug-treated rats and their vehicle-treated counterparts (data not shown).

### Adrenoreceptor expression during progression of TMJ degenerative remodelling

Condylar cartilage specimens from control rats had rich, even distribution of proteoglycans, with regularly-aligned subchondral bone (Supplemental Fig. 1A). Degenerative remodelling, characterised by cartilage degradation and subchondral bone loss, were evident in condyles of 4- and 8-wk experimental rats (Supplemental Fig. 1A). Evidence included decreased cartilage thickness and percentage area of proteoglycans (Supplemental Fig. 1B–D), decreased BMD, BV/TV, Tb.Th, and increased Tb.Sp in the experimental rats compared to age-matched controls (all p < 0.05; Supplemental Fig. 1E).

**Figure 1:**
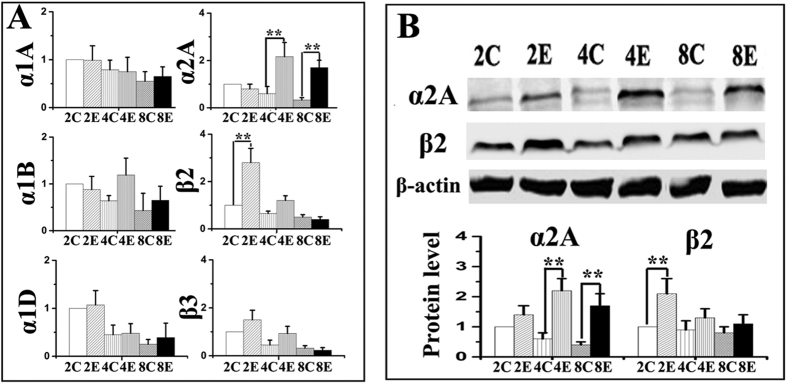
Real-time PCR (**A**) and western blot (**B**) analysis of the expression of different adrenoreceptors in the condylar cartilage from 2-, 4- and 8-wk control (C or Con) and experimental (E or Exp) rats (N = 5). Levels of significance for all charts: *P < 0.05, **P < 0.01: *vs* age-matched controls.

**Figure 2:**
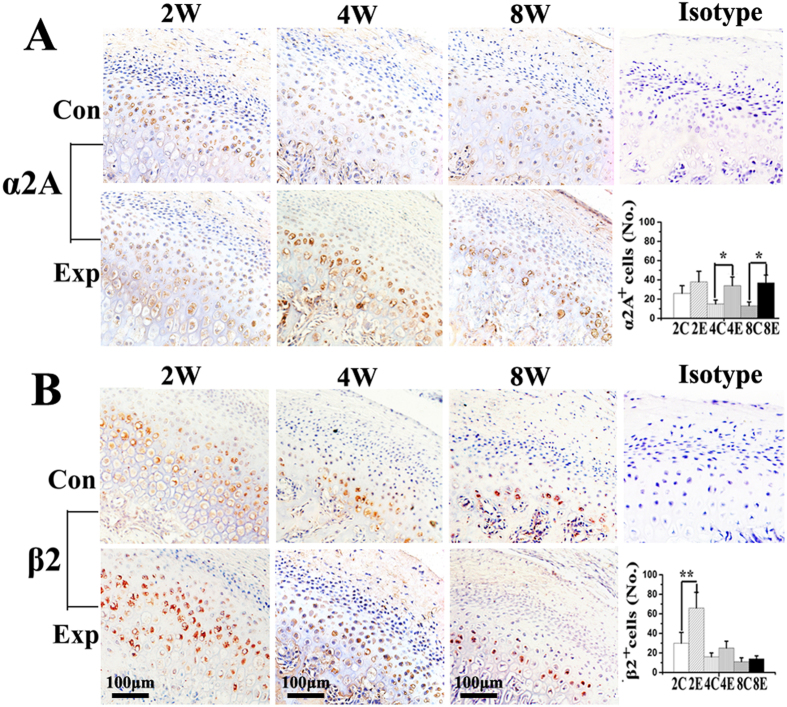
Immunohistochemical staining and quantification of α2A- and β2-adrenoreceptor positive ( + ) cells in the condylar cartilage in 2-, 4- and 8-wk control and experimental rats (N = 6). Levels of significance for all charts: *P < 0.05, **P < 0.01: *vs* age-matched controls.

The mRNA expressions of α1A-, α1B-, α1D-, α2A-, β2- and β3-adrenoreceptors were detected in all condylar cartilage examined (Fig. 1A), while those of α2B-, α2C- and β1-adrenoreceptors were not detected in any cartilage sample. Increased mRNA and protein expression of α2A-adrenoreceptor was observed in 4- and 8-wk experimental groups compared to age-matched controls, while increased expression of β2-adrenoreceptor was only observed in the 2-wk experimental groups (all p < 0.05; Fig. 1A,B). In control groups, α2A- and β2-adrenoreceptor positive chondrocytes were observed in all cartilage layers (Fig. 2A,B). In the experimental groups, the immune-signals of α2A- and β2-adrenoreceptor positive chondrocytes seemed more evident in the pre-hypertrophic and hypertrophic cartilage layers adjacent to osteochondral interface (Fig. 2A,B). Increased number of α2A-adrenoreceptor positive chondrocytes were observed in the condylar cartilage of 4- and 8-wk experimental rats, while increased β2-adrenoreceptor positive chondrocytes were only observed in 2-wk experimental rats, compared to age-matched groups (all p < 0.05; Fig. 2A,B). These results thus indicate that α2A- and β2-adrenergic signal transduction might be involved in the degenerative remodelling of condylar cartilage.

### Effects of norepinephrine and adrenoreceptors on chondrocyte pro-catabolic activities

**Figure 3:**
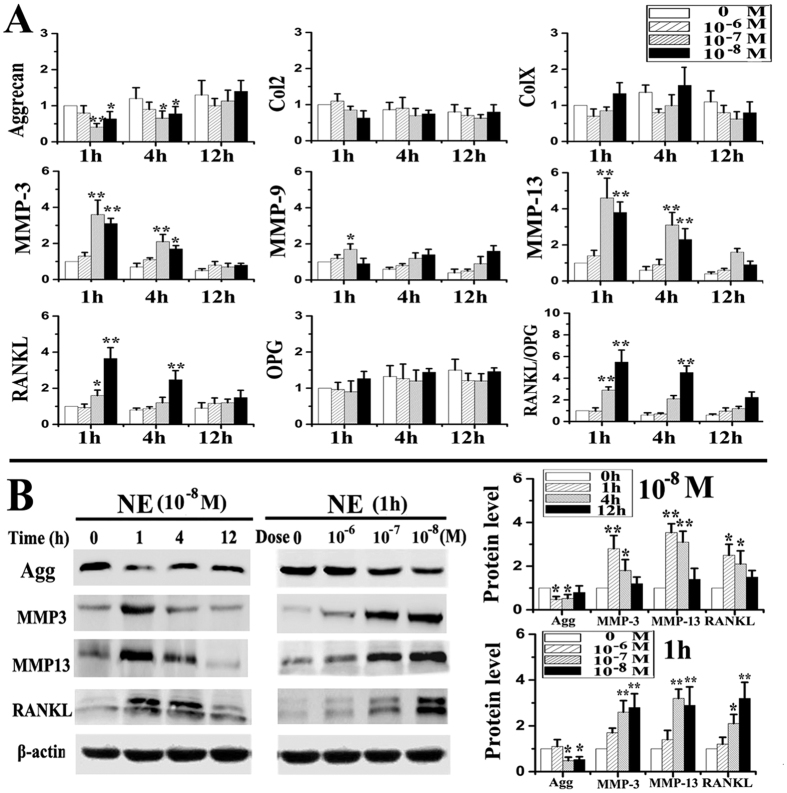
Norepinephrine decreased aggrecan expression, but increased expression of MMP-3, MMP-13 and RANKL. (**A**) Real-time PCR of the expression of aggrecan, type II collagen (Col2), type X collagen (ColX), MMP-3, MMP-9, MMP-13, RANKL, OPG and RANKL/OPG ratio by chondrocytes after NE stimulation (N = 5). The chondrocytes were isolated from condylar cartilage of 6-week female rats, and were treated with NE for 1, 4 or 12 h at 10^−6^, 10^−7^ or 10^−8^ M, respectively. (**B**) Western blot of the expression of aggrecan, MMP-3, MMP-13 and RANKL by chondrocytes after NE stimulation (N = 3). The chondrocytes were treated with 10^−8^ M NE for 1, 4 or 12 h, and treated 1h by 10^−6^, 10^−7^ or 10^−8^ M of NE, respectively. *P < 0.05, **P < 0.01: *vs* vehicle-treated chondrocytes.

**Figure 4:**
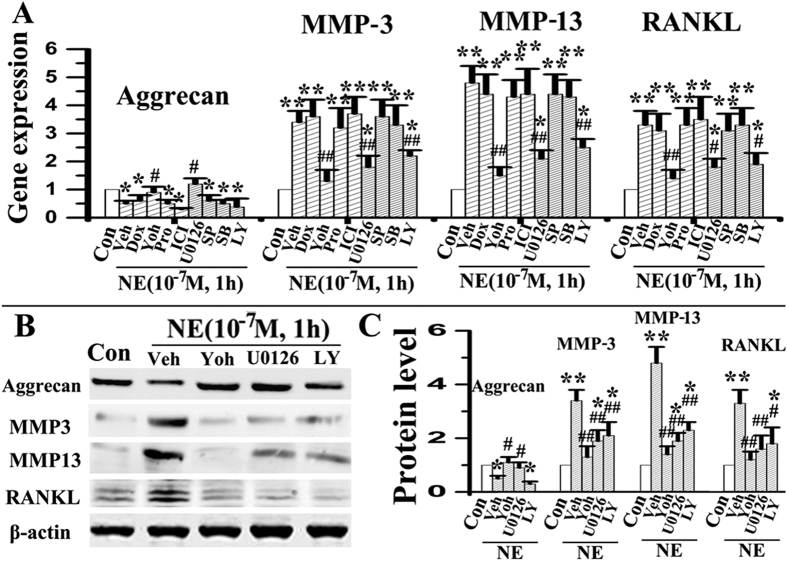
α2A-adrenoreceptor mediates the short-term pro-catabolic effects of norepinephrine. (**A**) Real-time PCR of the expression of aggrecan, MMP-3, MMP-13 and RANKL by chondrocytes after NE stimulation combined with different inhibitors of adrenoreceptors and pathways (N = 5). Chondrocytes were stimulated by 10^−7^ M NE for 1 h alone, or pre-treated for 1 h with 10^−5^ M doxazosin (Dox, α1-adrenoreceptor antagonist), yohimbine (Yoh, α2-adrenoreceptor antagonist), propranolol (Pro, β-adrenoreceptor antagonist), ICI 118,551 (ICI, β2-adrenoreceptor antagonist), U0126 (ERK1/2 inhibitor), SP600125 (SP, JNK inhibitor), SB203580 (SB, p38 inhibitor) or LY294002 (LY, AKT inhibitor). (**B,C**) Western blot of the expression of aggrecan, MMP-3, MMP-13 and RANKL by chondrocytes after NE stimulation combined with yohimbine (Yoh), U0126 or LY294002 (LY) treatment (N = 3). *P < 0.05, **P < 0.01: *vs* vehicle-treated chondrocytes; ^**#**^P < 0.05, ^**##**^P < 0.01: *vs* the corresponding NE-treated chondrocytes.

**Figure 5:**
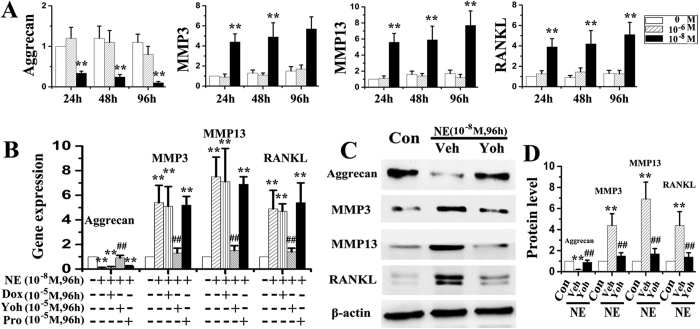
α2A-adrenoreceptor mediates the long-term pro-catabolic effects of norepinephrine. (**A**) Real-time PCR of the expression of aggrecan, MMP-3, MMP-13 and RANKL by chondrocytes after norepinephrine stimulation (N = 5). The chondrocytes were isolated from condylar cartilage of 6-week female rats, and were treated with norepinephrine for 24 h, 48 h or 96 h at 10^−6^ or 10^−8^ M, respectively. (**B**) Real-time PCR of the expression of aggrecan, MMP-3, MMP-13 and RANKL by chondrocytes after norepinephrine stimulation combined with different inhibitors of adrenoreceptors (N = 5). Chondrocytes were treated 96 hours by physiological slaine vehicle, 10^−8^ norepinephrine, or 10^−8^ norepinephrine in conjunction with 10^−5^ M doxazosin (Dox, α1-adrenoreceptor antagonist), yohimbine (Yoh, α2-adrenoreceptor antagonist) or propranolol (Pro, β-adrenoreceptor antagonist). (**C–D**) Western blot of the expression of aggrecan, MMP-3, MMP-13 and RANKL by chondrocytes after NE stimulation combined with yohimbine (Yoh) treatment (N = 3). **P < 0.01: *vs* vehicle-treated chondrocytes; ^**##**^P < 0.01: *vs* the corresponding NE-treated chondrocytes.

Almost all chondrocytes used for *in vitro* studies were positive for aggrecan and type II collagen, but were negative for type I collagen (Supplemental Fig. 2). When chondrocytes were stimulated with norepinephrine once, decreased mRNA expression of aggrecan, and increased expression of MMP-3, MMP-13, RANKL and RANKL/OPG were observed after 1 and 4 h of norepinephrine stimulation at 10^−7^ or 10^−8^ M (all p < 0.05, Fig. 3A). Gene expression of type II collagen, type X collagen, MMP-9 and OPG did not exhibit any difference between the norepinephrine-treated groups and vehicle-treated counterparts (all p > 0.05; Fig. 3A). For protein expression, decreased level of aggrecan, and increased levels of MMP-3, MM-P13 and RANKL were observed after 1 and 4 h of norepinephrine stimulation at 10^−8^ M, and after 1 h of stimulation at 10^−7^ and 10^−8^ M (all p < 0.05; Fig. 3B). There were no significant differences in the mRNA and protein expression of the aforementioned parameters between the norepinephrine-treated groups and vehicle-treated counterparts when norepinephrine was administered at 10^−6^ M or after 12 h (all p > 0.05, Fig. 3A,B). Furthermore, only the α2-adrenoreceptor antagonist yohimbine attenuated norepinephrine-induced increase in chondrocyte catabolic activities (aggrecan, MMP-3, MMP-13 and RANKL) at the gene (Fig. 4A) and protein levels (all p < 0.05; Fig. 4B,C). Other adrenoreceptor antagonists (α1, β and β2) had no reversal effects on those norepinephrine-induced changes (all p > 0.05; Fig. 4A). When chondrocytes were stimulated multiple times with norepinephrine, decreased mRNA expression of aggrecan, and increased expression of MMP-3, MMP-13 and RANKL were observed after 24, 48 and 96 h of norepinephrine stimulation at 10^−8^ M (all p < 0.05), but not at 10^−6^ M (all p > 0.05, Fig. 5A). Consistent with the results of short-term inhibition, only the α2-adrenoreceptor antagonist yohimbine attenuated the long-term pro-catabolic effects (aggrecan, MMP-3, MMP-13 and RANKL) of norepinephrinet at the gene (Fig. 5B) and protein levels (all p < 0.05; Fig. 5C), while other adrenoreceptor antagonists (α1 and β) had no reversal effects on those norepinephrine-induced changes (p > 0.05; Fig. 5B). Without concurrent NE treatment, adrenergic antagonists had no obvious effects on the above norepinephrine-induced increase in chondrocyte catabolic activities (data not shown). In line with above results, α2-adrenoreceptor agonist clonidine decreased mRNA expression of aggrecan by chondrocytes, and increased their expression of MMP-3, MMP-13 and RANKL (all p < 0.05, Supplemental Fig. 3), while yohimbine, isoprenaline or propranolol treatment have no significant effects on the expression of the aforementioned parameters by chondrocyte (all p > 0.05; Supplemental Fig. 3). Taken together, these results suggested that α2A-adrenoreceptor mediates the pro-catabolic effects of norepinephrine.

### Signalling pathways involved in norepinephrine-induced chondrocyte pro-catabolic activities

**Figure 6:**
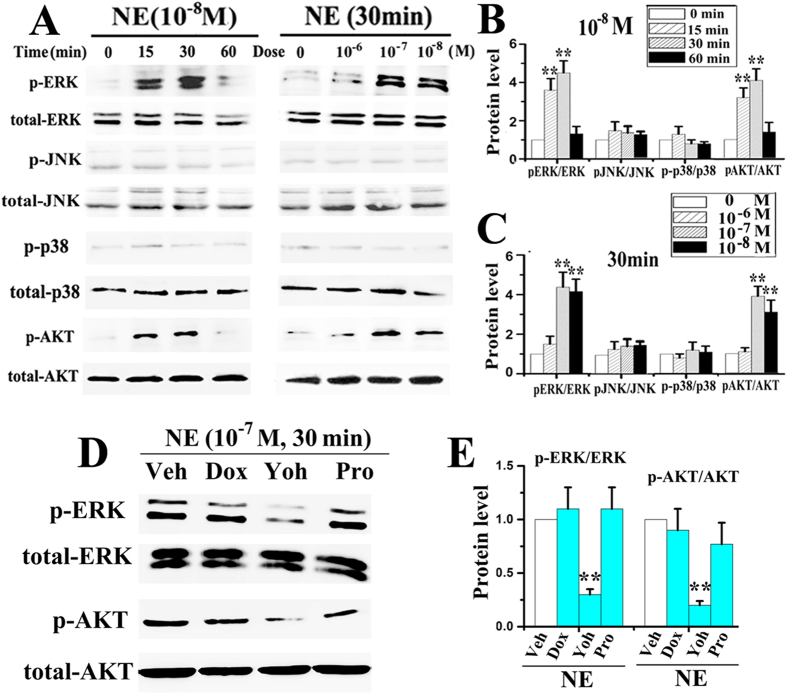
α2A-ERK/AKT axis mediates the pro-catabolic effects of norepinephrine. (**A–C**) Western blot of the expression of p-ERK, total ERK, p-JNK, total JNK, p-p38, total p38, p-AKT and total AKT by chondrocytes after NE stimulation (N = 3). The chondrocytes were treated by 10^−8^ M NE for 15, 30 or 60 min, and treated 30 min by NE of 10^−6^, 10^−7^ or 10^−8^ M. (**D,E**) Western blot of the expression of p-ERK, total ERK, p-AKT and total AKT by chondrocytes after NE stimulation in accompanied with different adrenorecptors antagonist (N = 3). Chondrocytes were stimulated by 10^−7^ M NE for 30 min alone, or pre-treated for 1 h with 10^−5^ μM doxazosin (Dox), yohimbine (Yoh) or propranolol (Pro). **P < 0.01: *vs* NE-treated chondrocytes.

Increases in p-ERK and p-AKT expression were appreciably detected after 15 and 30 min of norepinephrine stimulation at 10^−8^ M (Fig. 6A–C), and were statistically significant after 30 min of stimulation at 10^−7^ and 10^−8^ M (all p < 0.05; Fig. 6A–C). When norepinephrine was administered at 10^−6^ M or for 60 min, p-ERK and p-AKT expressions were not significantly different from the vehicle-treated counterparts (all p > 0.05; Fig. 6A–C). Expressions of p-p38 and p-JNK in the norepinephrine stimulation groups and the vehicle-treated counterparts were not significantly different at all time points or norepinephrine concentrations (all p > 0.05; Fig. 6A). In support of these observations, the ERK1/2 inhibitor U-0126 or Akt inhibitor LY294002 both significantly suppressed norepinephrine-induced increases in MMP-3, MMP-13 and RANKL expression at the gene (Fig. 4A) and protein levels (all p < 0.05; Fig. 4A–C); neither the p38 inhibitor SB203580 nor JNK inhibitor SP600125 had any blocking effect on the gene expression of those pro-catabolic factors (all p > 0.05; Fig. 4A). In line with these above results, the α2-adrenoreceptor antagonist yohimbine blocked norepinephrine-induced phosphorylation of ERK and AKT (all p < 0.05) while the other classes of antagonists (α1, β and β2) had negligible blocking effects (all p > 0.05; Fig. 6D,E). Taken together, these results indicated that norepinephrine-α2A signals in chondrocytes play a key role in the degenerative remodelling of condylar cartilage through the ERK1/2 and PKA pathways.

### Contribution of α2A-adrenoreceptors to condylar cartilage and subchondral bone deterioration

**Figure 7:**
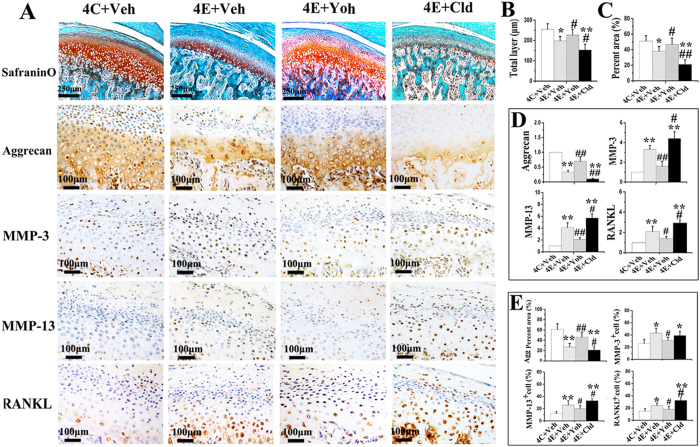
α2A-AR signalling mediates the severity of condylar cartilage degradation in TMJ-osteoarthritis by decreasing aggrecan expression and increasing MMP-3, MMP-13 and RANKL expression. Cartilage degradation was observed by H&E and Safranin O-fast green staining (**A**) in 4-wk control rats and experimental rats which were injected with vehicle saline (Veh), yohimbine (Yoh, α2-adrenoreceptor antagonist) or clonidine (Cld, α2-adrenoreceptor agonist), and the thickness and percentage area of proteoglycans of the condylar cartilage were compared ((**B,C**), N = 6). Data of real-time PCR (**D**), N = 5) and immunohistochemical staining (**A**) and quantification ((**E**), N = 6) of the expression of aggrecan, MMP-3, MMP-13 and RANKL in above condylar cartilage samples were shown. C: control rats; E: experimental rats. *P < 0.05, **P < 0.01: *vs* vehicle-treated control rats; ^**#**^P < 0.05, ^**##**^P < 0.01: *vs* vehicle-treated experimental rats.

**Figure 8:**
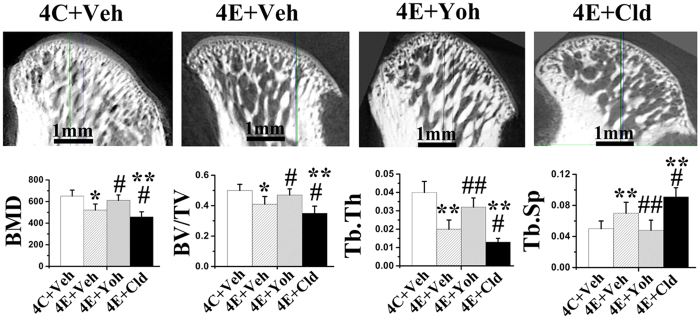
α2A-adrenoreceptor mediates the severity of condylar subchondral bone deterioration in TMJ-osteoarthritis. Micro-CT was used to observed the bone mineral density (BMD) and microstructures of condylar subchondral bone from 4-wk control rats (4C) and experimental rats (4E) which were injected with vehicle saline (Veh), yohimbine (Yoh, α2-adrenoreceptor antagonist) or clonidine (Cld, α2-adrenoreceptor agonist) (N = 6). **P < 0.05, **P < 0.01: *vs* vehicle-treated control rats; ^**#**^P < 0.05, ^**##**^P < 0.01: *vs* vehicle-treated experimental rats.

Blocking of α2A-adrenoreceptor by yohimbine greatly attenuated the progression of cartilage degenerative changes in experimental rats, as evidenced by the increased cartilage thickness and percentage area of proteoglycans and aggrecan (Fig. 7A–C), and decreased mRNA and protein expression of MMP3, MMP13 and RANKL in condylar cartilage specimens (Fig. 7A,D,E), increased BMD, BV/TV, Tb.Th, as well as decreased Tb.Sp in subchondral bone (Fig. 8), when compared to the saline vehicle-treated experimental rats (all p < 0.05). Conversely, activation of α2A-adrenoreceptor by clonidine further intensified cartilage degradation and subchondral bone deterioration in experimental rats when compared to the saline vehicle-treated experimental rats, as revealed by decreased cartilage thickness and percentage area of proteoglycans and aggrecan, and increased mRNA and protein expression of MMP13 and RANKL in cartilage (all p < 0.05; Fig. 7), decreased BMD, BV/TV, Tb.Th, and increased Tb.Sp in the subchondral bone (all p < 0.05; Fig. 8). Conversely, blocking of β2-adrenoreceptor by propranolol had no significant rescuing effects on cartilage thickness and proteoglycans distribution in experimental rats comparing to the vehicle-treated counterparts (all p > 0.05; Supplemental Fig. 4).

## Discussion

In the present study, degenerative cartilage remodelling and subchondral bone loss was accompanied by increased gene and protein expressions of α2A-adrenoreceptor in condylar cartilage of UAC rats. Inhibition of this receptor attenuated degenerative remodellings of cartilage and subchondral bone, while activation of these receptors intensified those degenerative remodellings. Mechanism wise, activation of norepinephrine-α2A signals in chondrocytes via the ERK1/2 and PKA pathways stimulated production of factors involved in osteochondral complex degradation, including MMP-3, MMP-13 and RANKL. These observations were supported by *in vitro* and *in vivo* evidence, in that blocking of α2A-adrenoreceptor increased aggrecan production and decreased pro-catabolic factor expressions in norepinephrine-stimulated chondrocytes and in the degraded cartilage of UAC rats. Taken together, the data indicate that α2A-adrenergic signal transduction in chondrocytes plays a detrimental role in the pathologenesis of TMJ degenerative remodelling, while inhibition of α2A-adrenergic signal attenuates TMJ degenerative remodelling.

Increased expressions of β2-adrenoreceptor in the condylar cartilage of 2-week experimental rats may represent the response of chondrocytes to counteract degenerative remodeling and to restore the degenerative cartilage^13^. It has been known that β2-adrenoreceptor signaling plays an anti-inflammatory role in the pathogenesis of osteoarthritis and promotes a non-proliferative, metabolically-stable articular chondrocyte phenotype which may counteract osteoarthritis initiation or progression^13^. However, this form of compensation is generally unsuccessful over time, partially because increased β2-adrenoreceptor expression by chondrocytes ended at the 4-week experimental period. Furthermore, the protective effect by β2-adrenoreceptor signaling is limited because injection of the β-adrenoreceptor agonist isoprenaline into the TMJ region of UAC rats did not reverse degenerative changes in the cartilage during the observation period. Conversely, increased α2A-adrenoreceptor expression occurred at the 4-week and 8-week experimental periods. The pathogenic roles of α2A-adrenergic signaling in the degenerative remodeling of cartilage were supported by three important cues. First, among all the adrenoreceptors expressed by chondrocytes, only α2A-adrenoreceptor expression increased in the condylar cartilage of 4- and 8-week experimental rats, in conjunction with degenerative cartilage remodeling and subchondral bone loss. Second, the effects of norepinephrine on chondrocyte expression of aggrecan, MMPs and RANKL were most apparent when its concentration was 10^−8^ M. These changes were progressively attenuated as the concentration of norepinephrine increased, with no effects observed when the norepinephrine concentration was increased to 10^−6^ M. At low concentrations (≤10^−7^ M), norepinephrine is mediated mainly via α-adrenoreceptors, whereas at high concentrations (>10^−7^ M), norepinephrine act preferentially via the β-adrenoreceptors^30^. Hence, the pro-catabolic changes induced by norepinephrine on adult chondrocytes are likely to be mediated by α-adrenergic signaling. This is further supported by the observation that only the α2A-adrenoreceptor antagonist yohimbine could reverse norepinephrine-induced expression of pro-catabolic mediators by chondrocytes, while antagonists of the other adrenoreceptors lacked those reversal effects. Third, blocking of α2A-adrenoreceptor in local TMJ regions of UAC rats increased cartilage thickness and proteoglycans distribution in the condylar cartilage, and decreased expression of pro-catabolic mediators by chondrocytes, while activation of this receptor intensified those indices of cartilage degradation. The pro-catabolic role of α2A signaling during degenerative remodeling of cartilage was in accordance with previous studies, in that norepinephrine accelerates hypertrophic differentiation of chondrocytes in patients with osteoarthritis^4^. In addition, absence of sympathetic nerves delays hypertrophic differentiation of chondrocytes in the callus of fractures bones developed by endochondral ossification^31^. Apart from the pathomechanism described here, the pathogenic effect of α2-adrenergic signaling has also been shown to promote osteoarthritis progression through induction of apoptosis of articular chondrocytes^13^.

The authors have previously reported that activation of β2-adrenoreceptor signaling has a detrimental effect on condylar subchondral bone loss during TMJ degradation, and ectogenic activation of β2-adrenoreceptor by intraperitoneal injections of isoproterenol further intensified degenerative cartilage remodelling in unilateral anterior crossbite (UAC) rats^22^. In the present work, although the difference was not statistically significant, we also observed that local injection of isoproterenol into the TMJ intensified degenerative cartilage remodelling in UAC rats. However, our previous and present data showed that neither intraperitoneal injection nor local TMJ injection of β-antagonist (propranolol) could rescue degenerative cartilage remodelling in the UAC rats. Combined with the present *in vitro* data that the β-antagonist did not block the pro-catabolic effects of norepinephrine on chondrocytes, these data suggest that the detrimental effect of β2-adrenoreceptor signaling on TMJs could have resulted from its pathogenic effects on bone cells^22^. Further study using mice with α2A- or β2-adrenoreceptor conditional knockout from chondrocytes, by mating α2A- or β2-adrenoreceptor Flox/Flox mice with type II collagen-Cre transgenic mice, are needed to further clarify signaling from adrenoreceptors in cartilage degradation.

Although α2-adrenoreceptor signalling inhibits adenylyl cyclase and voltage-gated calcium channels via activation of the MAPK and AKT pathways^32^,^33^,^34^,^35^,^36^,^37^, signal transduction of α2A-adrenoreceptor in chondrocytes remains unclear. The present study showed that norepinephrine activated ERK1/2 and AKT pathways in adult chondrocytes in a dose- and time-dependent manner, but did not activate the JNK or p38 pathway with the doses and stimulation times employed. Moreover, the use of an α2A-adrenoreceptor antagonist fully abolished ERK1/2 and AKT activation, while the use of an ERK1/2 or AKT inhibitor partially reversed MMP-3, MMP-13 and RANKL expressions by chondrocytes. These novel results are consistent with previous studies showing that the pro-absorptive effects of norepinephrine on intestinal epithelial cells are mediated by ERK1/2 and AKT pathways^38^, and that activation of those pathways mediates upregulation of MMPs by chondrocytes^39^,^40^ and RANKL by osteoblasts^41^,^42^. Our novel observation that only the ERK1/2 inhibitor could rescue norepinephrine-induced aggrecan production by chondrocytes is in par with the previous observation that TNF-α induced reduction in aggrecan expression by chondrocytes mainly mediated by MEK/ERK signalling^43^.

There is ample evidence demonstrating that β2-adrenergic signalling suppresses bone formation and promotes bone resorption^3^. Interestingly, the present data shows the similar effects of α2A-adrenoreceptor signalling on bone, as evidenced by the fact that blocking α2A-adrenoreceptor in UAC rats rescued their condylar subchondral bone loss, while activation the α2A-adrenoreceptor further intensified their subchondral bone loss. This result may be explained by the pro-osteoclastic effect of α2A-adrenergic signalling via promotion of osteoclast maturation; α2A-adrenoreceptor global knockout mice exhibited high bone mass in their femur and tibia^44^. In addition, hypertrophic chondrocytes adjacent to the osteochondral interface of osteoarthritic cartilage expressed high levels of pro-osteoclastic factors^18^,^19^ that could permeate the subchondral bone and promote bone loss following breaching of the osteochondral barrier in progressive osteoarthritis^45^,^46^. Data derived from the present study showed increased expression of α2A-adrenoreceptor in hypertrophic chondrocytes near the osteochondral interface of degraded cartilage. Moreover, activation of α2A-adrenergic signalling enhanced the expression of pro-osteoclastic MMP-13 and RANKL. Further co-culturing studies using norepinephrine-stimulated chondrocytes and pre-osteoclasts are required to clarify the role of α2A-adrenergic signalling on chondrocyte-mediated osteoclast recruitment, development and bone resorption.

One may expect that sympathetic nervous system plays pathogenic roles in the progression of the degenerative remodelling of cartilage and subchondral bone. The authors have recently shown that extrinsic augmentation of the sympathetic tone in UAC mice by chronic immobilisation stresses aggravated condylar cartilage degradation and subchondral bone loss^22^. Although sympathetic denervation of those TMJs by intraperitoneal injection of 6-hydroxydopamine reduced their norepinephrine levels in blood and condylar subchondral bone and rescued subchondral bone loss, such a procedure had negligible effect on reversing the degradation of condylar cartilage^22^. Because norepinephrine may be produced by different catecholamine-producing cells in the synovial tissues of patients with osteoarthritis or rheumatoid arthritis^47^, there may be other origins of norepinephrine in the degraded TMJ joint apart from the presence of sympathetic nerve fibres. These catecholamine-producing cells may switch-on the norepinephrine-producing machinery as an endogenous compensatory mechanism for the loss of sympathetic nerve fibres during the progression of rheumatoid arthritis^47^. Thus, the role of synovial tissue-derived norepinephrine on osteoarthritis progression should be investigated in future work.

Although the cartilage specimens used for chondrocyte isolation, PCR and western blot were carefully dissected under a dissecting microscope to exclude bone tissues, it is difficult to avoid contamination from bone cells based on the intimate nature of the TMJ bone/cartilage interphase. Following the isolation and culture protocol we and others have reported^21^,^25^,^48^,^49^, almost all condyle chondrocytes used for the *in vitro* part of the present study were positive for aggrecan and type II collagen and negative for type I collagen. In addition, agreement of the PCR and western blot results with immunohistochemical quantification of cartilage also partially support that the specimens examined here are predominantly cartilageous. Nevertheless, further studies using mice with α2A- or β2-AR conditional knockout from cartilage and/or collagen II positive cells sorted by flow cytometry are needed to confirm the present findings. Another issue that requires clarification is that there is aunique fibrous layer on the superficial zone of the condylar cartilage which significantly reduces its deformation of condylar cartilage under compression and the influence of surface frictional forces^50^. Some reports showed that this fibrous layer containing fibroblasts that can synthesize type I collagen^50^,^51^. However, all of the cells we currently isolated from condylar cartilage were positive for toluidine blue, aggrecanand type II collagen staining, but not for type I collagen. This is in agreement with the results of Klinge^52^ and Landesberg^53^, who reported that all four layers of the condylar cartilage are composed of chondrocytes and those cells in the superficial zone are fibroblast-like chondrocytes. Various factors can stimulate fibroblast-like chondrocytes to synthesize type I collagen *in vivo* to protect condylar cartilages that are subjected to compression and friction forces. Due to lack of force stimulation *in vitro*, those chondrocytes do not synthesize type I collagen^54^. Therefore, the primary condylar cells used in the present study appropriately reflect the response of condylar chondrocytes to norepinephrine. Because the objective of the present study was not to demonstrate the physiologic and/or pathologic roles of adrenoreceptor-mediated signaling in the TMJ, further studies are required to establish the distribution profiles of endogenous ligand(s) for these receptors in early and advanced osteoarthritic conditions. Given the fact that the expression profiles of adrenoreceptors change during TMJ-osteoarthritis progression, it is important in future studies to examine condylar chondrocytes from different osteoarthritic stages with norepinephrine stimulation.

In conclusion, the present study demonstrates, for the first time, that activation of α2A-adrenergic signal transduction in chondrocytes by norepinephrine promotes degenerative remodelling of condylar cartilage and loss of subchondral bone in rats induced by abnormal loading. Signal transduction via activation of the ERK and AKT pathways exacerbates chondrocyte-mediated pro-catabolic activities during the progression of TMJ degenerative remodelling. Inhibition of α2A-adrenoreceptor signal thus offers a novel therapeutic target for inhibition/reversal of degenerative remodelling of TMJ.

## Additional Information

**How to cite this article**: Jiao, K. *et al*. Activation of α2A-adrenergic signal transduction in chondrocytes promotes degenerative remodelling of temporomandibular joint. *Sci. Rep.* **6**, 30085; doi: 10.1038/srep30085 (2016).

## Supplementary Material

Supplementary Information
