# Supplementary material for: Activation of α2A-adrenergic signal transduction in chondrocytes promotes degenerative remodelling of temporomandibular joint
Source: Sci Rep. 2016 Jul 25;6:30085. doi: 10.1038/srep30085 (PMC4958971; doi:10.1038/srep30085)
Supplement: Supplementary Information [file srep30085-s1.pdf]

Activation of α2A-adrenergic signal transduction in chondrocytes promotes degenerative remodelling of temporomandibular joint

Kai Jiao[1,#], Guang Zeng[2,#], Li-Na Niu[3], Hong-xu Yang[1], Gao-tong Ren[4], Xin-yue Xu[4], Fei-fei Li[5], Franklin R. Tay[6,*], Mei-qing Wang[1,*]

Supplementary Table1 Gene primers

Genes	Forward primer	Reverse primer
α1A-AR	GAGTCAGCAGTGCCAAGAATAAGA	AACGGTTTCCGAAGGCTTGA
α1B-AR	CCCAGGAGTTCCATAGCTGTCAA	TGAAGTAGCCCAGCCAGAACAC
α1D-AR	CCATCGTCGTGGGTGTCTTC	GTAGATGAGCGGGTTCACACAG
α2A-AR	CTTGGCCCTCGACGTGCTCTTTTG	CGGCGGCGCGGAACAGG
α2B-AR	TCGGCCATCACCTTTCTCATCCTT	GGGCGTCGGGGCGTTGGTC
α2C-AR	TGGCTCATCTCGGCTGTCATCTCC	TGTCCCCCTCGGCACCCTCTC
β1-AR	TCTGTGAGCTCTGGACTTCGGTA	GATGACACACAGGGTCTCGATG
β2-AR	GATTGCAGTGGATCGCTATGTTG	GACCACTCGGGCCTTATTCTTG
β3-AR	CCCTTTCTTCCTACTGCTTTCCT	TTTGTGCCTATTGTGAGAGATGGT
aggrecan	ATGATGGCGCTGTTCTGAAGG	GAAGTGATGCATGGCATTGAGG
Col2	ACCTCGGATTCCAATAGGACCAG	TTCCCGGTGAATTCGGTCTC
ColX	GGCTACTTGGATCAGGCTTC	CTGAGAAGGACGAGTGGACA
MMP3	TCTTTCACTCAGCCAATGCT	GGGAGGTCCATAGAGGGATT
MMP9	GAGCCACGACCATACAGATG	CGCTGGGCTTAGATCATTCT
MMP13	GCAGCTCCAAAGGCTACAA	CATCATCTGGGAGCATGAAA
RANKL	TCGGGTTCCCATAAAGTCAG	CTTGGGATTTTGATGCTGGT
OPG	TGGGAATGAAGATCCTCCAG	GAGGAAGGAAAGGGCCTATG
GAPDH	TGTGTCCGTCGTGGATCTGA	TTGCTGTTGAAGTCGCAGGAG

AR: Adrenoreceptor

Supplementary Figure 1

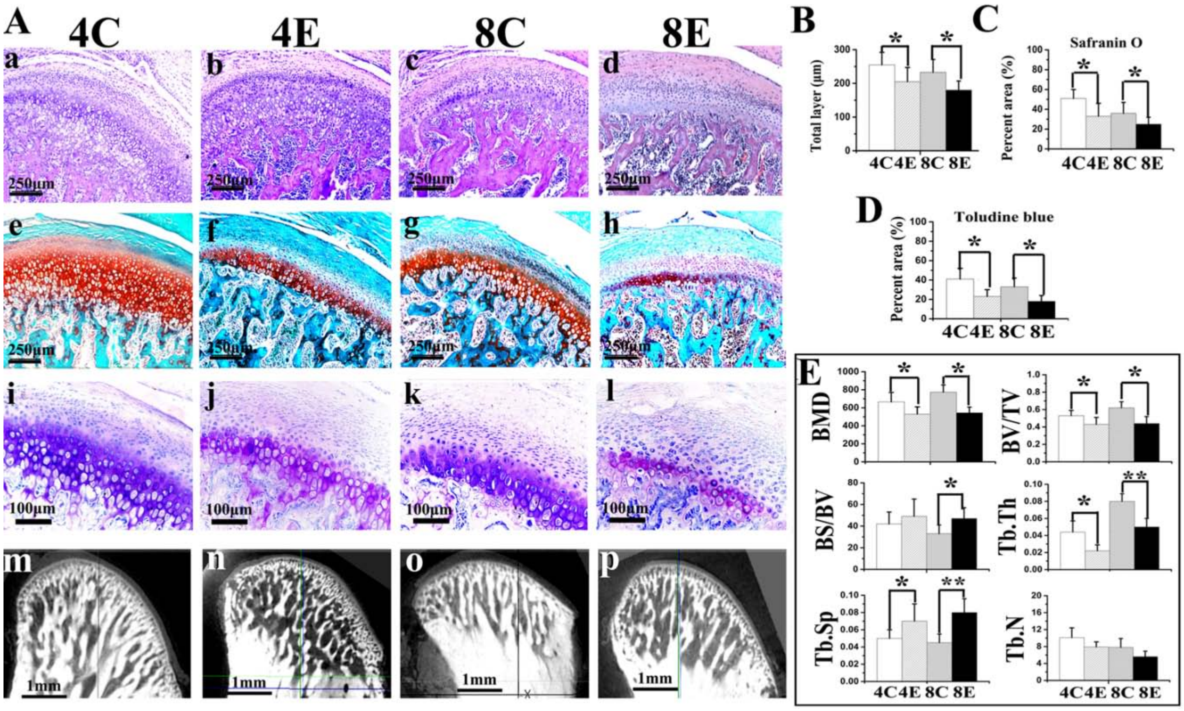

Supplementary Figure 1 Cartilage degeneration and subchondral bone deterioration observed in 4- and 8-week experimental rats. (A) Cartilage degeneration were observed by H&E (a-d), Safranin O-fast green (e-h) and toluidine blue (i-l) staining. Microarchitecture of subchondral bone was quantified by micro-computed tomography (m-p). (B-D) Thickness and percentage area of proteoglycans within the condylar cartilage of 4- and 8-week control and experimental rats (N=6). (E-H) Bone mineral density (BMD) and microstructures of subchondral condylar bone in the 4- and 8-week control and experimental rats (N=6). C: control rats; E: experimental rats. Levels of significance: *P<0.05, **P<0.01: *vs* age-matched controls.

Supplementary Figure 2

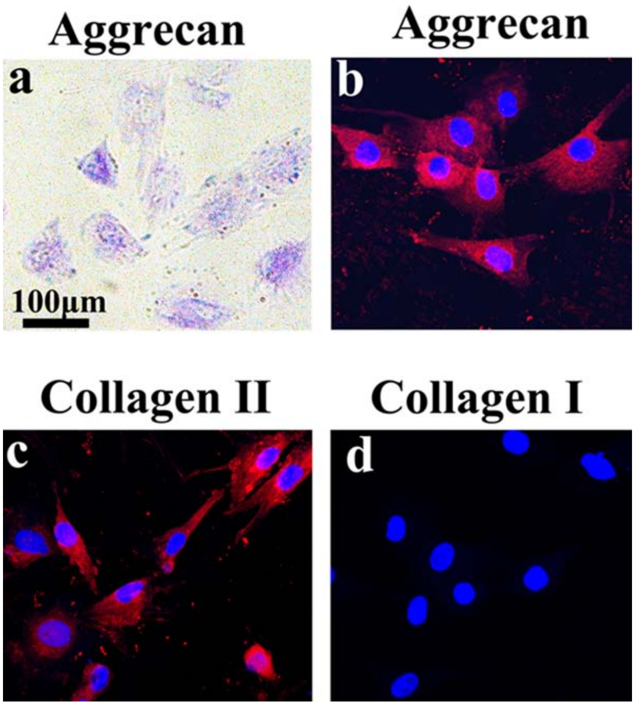

Supplementary Figure 2 Chondrocytes identification. Chondrocytes were identified by (a) toluidine blue staining and (b) immunofluorescent staining of aggrecan, and (c) type II and type I collagen.

Supplementary Figure 3

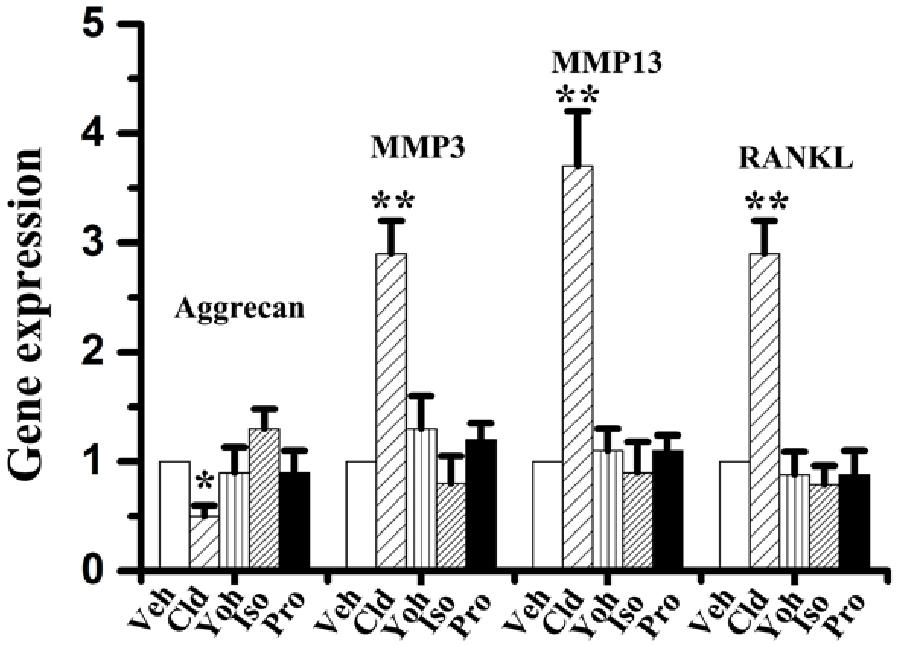

Supplementary Figure 3 Real-time PCR of the expression of aggrecan, MMP-3, MMP-13 and RANKL by chondrocytes treated by α2-adrenoreceptor agonist/antagonist or β2-adrenoreceptor agonist/antagonist (N=5). The chondrocytes were isolated from condylar cartilage of 6-week female rats and were stimulated 24 h by saline vehicle (Veh), 10^{-4} M clonidine (Cld, α2-adrenoreceptor agonist), 10^{-5} M yohimbine (Yoh, α2-adrenoreceptor antagonist), 10^{-6} M isoprenaline (Iso, β-adrenoreceptor agonist) or 10^{-5} M propranolol (Pro, β-adrenoreceptor antagonist). *P<0.05, **P<0.01: vs vehicle-treated controls.

Supplementary Figure 4

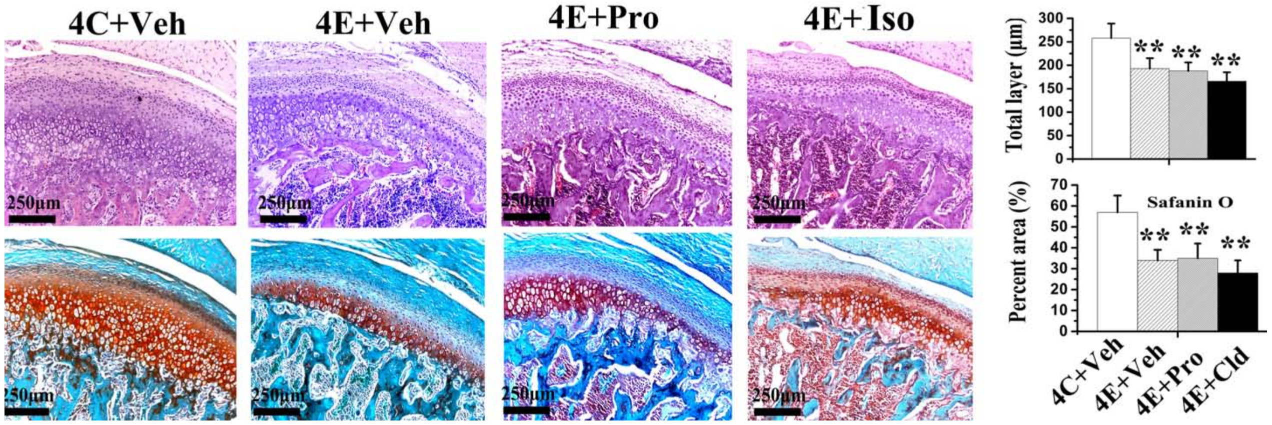

Supplementary Figure 4 Effect of blocking/activation of β2-adrenoreceptor on condylar cartilage degenerative remodelling. Cartilage degradation was observed by H&E and Safranin O-fast green staining in 4-week control rats and experimental rats which were injected with saline vehicle (Veh), propranolol (Pro, β2-adrenoreceptor antagonist) or isoprenaline (Iso, β2-adrenoreceptor agonist). The thickness and percentage area of proteoglycans of the condylar cartilage were compared (N=6). C: control rats; E: experimental rats. **P<0.01: *vs* vehicle-treated control rats.
